# Supplementary material for: Splice-Junction-Based Mapping of Alternative Isoforms in the Human Proteome
Source: Cell Rep. Author manuscript; Available in PMC 2020 Jan 15. (PMC6961840; doi:10.1016/j.celrep.2019.11.026)
Supplement: 2 [file NIHMS1546469-supplement-2.zip › DF1/PXD009737/TestisGluC/TestisGluC_14_MMAB_KGDPKMTKCLKPWELQMNLGLGSRLGLRGCFGAARL.pdf]

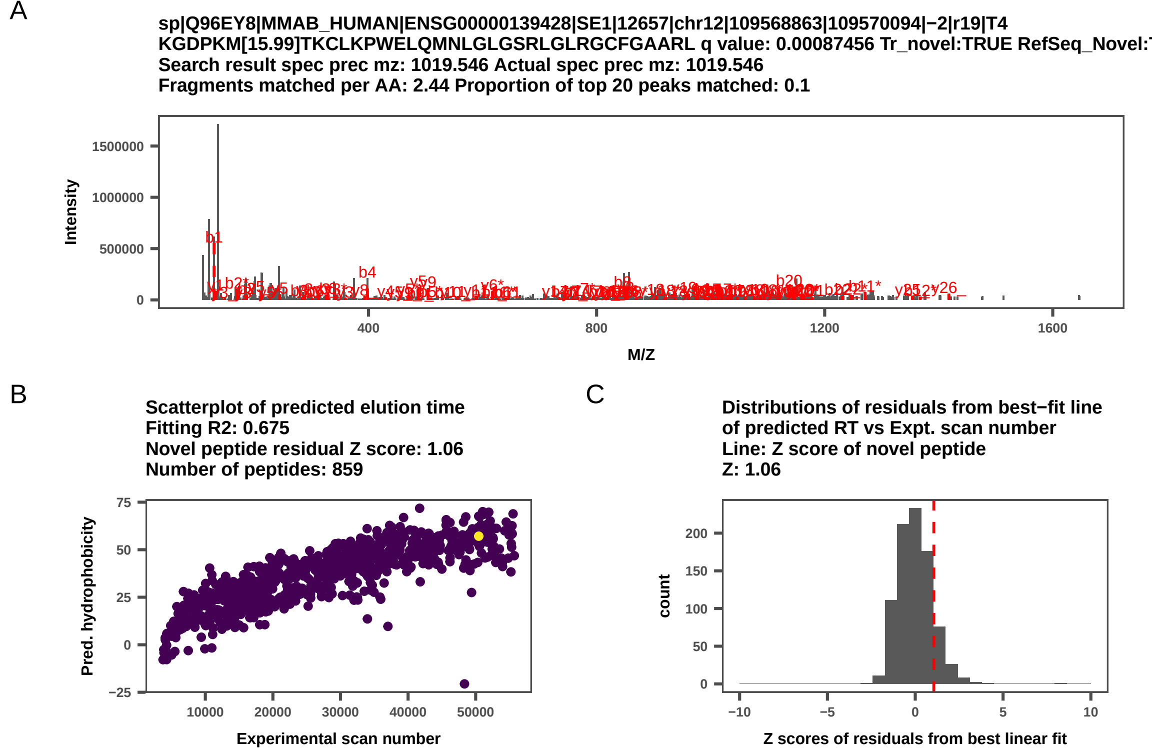
A
sp|Q96EY8|MMAB_HUMAN|ENSG00000139428|SE1|12657|chr12|109568863|109570094|−2|r19|T4
KGDPKM[15.99]TKCLKPWELQMNLGLGSRLGLRGCFGAARL q value: 0.00087456 Tr_novel:TRUE RefSeq_Novel:
Search result spec prec mz: 1019.546 Actual spec prec mz: 1019.546
Fragments matched per AA: 2.44 Proportion of top 20 peaks matched: 0.1
Intensity
1500000
1000000
500000
0
b1
b4
b20
y26
400
800
1200
1600
M/Z
B
Scatterplot of predicted elution time
Fitting R2: 0.675
Novel peptide residual Z score: 1.06
Number of peptides: 859
Pred. hydrophobicity
75
50
25
0
−25
10000
20000
30000
40000
50000
Experimental scan number
C
Distributions of residuals from best−fit line
of predicted RT vs Expt. scan number
Line: Z score of novel peptide
Z: 1.06
count
200
150
100
50
0
−10
−5
0
5
10
Z scores of residuals from best linear fit
